# Emerging roles of ATP citrate lyase in kidney diseases: from pathogenic driver to therapeutic target

**DOI:** 10.1080/0886022X.2026.2652826

**Published:** 2026-04-09

**Authors:** Mengjiao Wei, Mina Tao, Huifang Tan, Zhiyuan Jin, Yiya Yang, Zheng Xiao, Guoli Li, Yinyin Chen

**Affiliations:** a Department of Nephrology, Hunan Clinical Research Center for Chronic Kidney Disease, Hunan Provincial People’s Hospital, The First Affiliated Hospital of Hunan Normal University, Changsha, Hunan, China; b Hunan Engineering Research Center for Prevention, Treatment and Rehabilitation of Kidney Diseases, Changsha, Hunan, China; c Changsha Innovation Center for Integrated Diagnosis and Treatment Technology of Metabolic Dysfunction–Associated Kidney Diseases, Changsha, Hunan, China

**Keywords:** ATP citrate lyase, kidney diseases, lipid metabolism, histone acetylation, bempedoic acid

## Abstract

ATP-citrate lyase (ACLY) is a crucial cytosolic and nuclear enzyme that catalyzes the synthesis of acetyl-CoA from citrate, serving as a central metabolic node linking carbohydrate metabolism to *de novo* lipogenesis and histone acetylation. Accumulating evidence positions ACLY not merely as a metabolic enzyme but as a key pathogenic driver in various kidney diseases. This review systematically examines the multifaceted roles of ACLY in renal physiology and pathology. During development, ACLY is essential for nephron progenitor cell maintenance and nephrogenesis. In diabetic kidney disease, hyperglycemia upregulates and promotes nuclear translocation of ACLY, fueling histone acetylation and the transcription of pro-fibrotic genes, while also contributing to oxidative stress and lipid peroxidation. In obesity-related kidney injury, ACLY drives renal ectopic lipid accumulation and inflammation by providing substrates for adipogenic enzymes and inducing histone hyperacetylation. ACLY also promotes renal fibrosis in chronic kidney disease *via* pathways such as AKT/ACLY signaling. In clear cell renal cell carcinoma, ACLY expression is upregulated through HIF-2α/LPCAT1/FBXW7 and VHL/PPARγ axes, promoting lipid synthesis, tumor proliferation, and metastasis. Furthermore, ACLY activity influences hypocitraturia, a key factor in nephrolithiasis, and is upregulated in polycystic kidney disease, where its inhibition attenuates cystic growth. Given its central role across diverse renal pathologies, ACLY emerges as a promising therapeutic target. Several inhibitors, including bempedoic acid, SB-204990, and natural compounds, show potential in modulating ACLY activity. This review consolidates current knowledge on ACLY in kidney diseases, highlighting its mechanistic contributions, and underscoring its significant potential for diagnostic and therapeutic innovation.

## Introduction

Kidney disease is a major health issue worldwide, with incidence rates continuing to rise and often accompanied by high rates of disability and mortality [1,2]. Recent epidemiological data reveal that chronic kidney disease (CKD) affects over 800 million individuals worldwide, representing 8–16% of the global population [3]. Kidney disease leads to premature death, disability, reduced quality of life, social and psychological harm, and high medical costs [1]. Currently, clinical management of CKD and acute kidney injury (AKI) remains hindered by the absence of targeted therapies, largely due to an incomplete understanding of their underlying pathogenic mechanisms, which limits treatment efficacy [4]. Therefore, in-depth exploration of the molecular mechanisms of kidney disease and identification of new therapeutic targets hold significant clinical implications.

The kidneys, as important metabolic regulatory organs in the body, play a central role in maintaining material and energy balance. Various types of kidney damage, including diabetic kidney diseases (DKD) [5] and obesity related kidney diseases [6,7], are commonly associated with metabolic disorders, particularly abnormal lipid metabolism. The phenomenon of lipid accumulation in the kidney (‘fatty kidney’) was first reported in 1883[8,9]. The pathogenic *versus* consequential role of this fat remained contentious until subsequent experimental work has definitively shown that it causally contributes to renal fibrosis and the progression of CKD [8]. Increasing evidence suggests that alterations in lipid metabolism are not merely a concomitant phenomenon of kidney disease but actively participates in its onset and progression, influencing processes such as cell apoptosis, inflammatory responses, and fibrosis [10]. Disease-specific abnormalities in cholesterol and fatty acid metabolism may adversely affect kidney health and play a role in the development and progression of kidney disease [11]. Nevertheless, despite considerable advances in our understanding of renal lipid metabolism, numerous questions remain unresolved. ATP-citrate lyase (ACLY) is a key enzyme linking glucose metabolism to lipid synthesis by producing acetyl-CoA. And previous studies have revealed that multiple short-chain fatty acids in the kidneys participate in the progression of kidney disease at the metabolic-epigenetic level [12–15]. It plays a central role in fatty acid and cholesterol biosynthesis, impacting both physiology and disease [16]. Its overexpression is implicated in metabolic disorders, cancer, and also kidney disease, rendering it a promising therapeutic target [17,18]. Given this central role, this review aims to systematically elucidate the function of ACLY in renal lipid metabolism, delineate its underlying molecular mechanisms, and evaluate the therapeutic potential of targeting ACLY for the treatment of kidney diseases.

## Overview of ACLY

ACLY is a crucial enzyme in cellular metabolism that catalyzes the conversion of citrate and coenzyme A into acetyl-CoA and oxaloacetate, which can be detected in both the cytoplasm and nucleus [19]. It links carbohydrate and lipid metabolism by generating acetyl-CoA from citrate for fatty acid and cholesterol biosynthesis [16]. ACLY is structurally a tetrameric protein that consists of four subunits of 11,0000 Da each [20]. ACLY recognizes ATP, CoA, ADP, 3′-AMP and NADP, and has the highest affinity for CoA. ACLY could be activated by a variety of anions, such as chloride, bicarbonate and acetate ions [21]. Acetyl-CoA is a key substrate for protein acetylation, especially for histones [22]. ACLY catalyzes the production of acetyl-CoA is primarily located in the cytoplasm, where its core function is to catalyze the conversion of citrate into acetyl-CoA. Since acetyl-CoA itself cannot easily cross the nuclear membrane, a significant source of acetyl-CoA required for histone acetylation in the nucleus relies on the catalytic activity of ACLY present in the nucleus. Nuclear ACLY utilizes citrate, which can freely diffuse into the nucleus, as a precursor to synthesize acetyl-CoA *in situ* near chromatin. This mechanism directly regulates the local concentration of nuclear acetylation substrates, making ACLY a key rate-limiting factor for histone acetylation [23].

**Figure 1.**
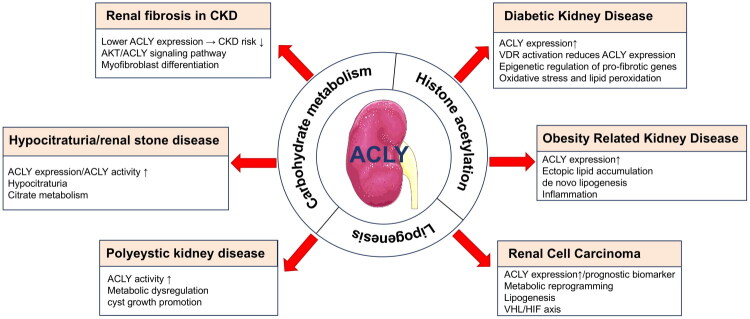
Overview of the role of ACLY in kidney diseases. CKD: chronic kidney disease; AKT- VDR- Vitamin D Receptor; VHL/HIF: Von Hippel–Lindau/Hypoxia Inducible Factor.

Recently, ACLY has gained increasing attention as both a promising diagnostic biomarker and a viable therapeutic target for multiple chronic disorders, such as neurodegenerative disease, cardiovascular disease, diabetes, obesity, inflammation, and cancer [24]. ACLY also plays a pivotal role in renal physiology and pathology (Figure 1 and Table 1) [25–27,35,36]. Emerging evidence, including our prior work, highlights its function as a critical regulator of renal ectopic lipid accumulation and fibrosis. Next, we will systematically discuss the detailed mechanistic role of ACLY in renal pathophysiology.

**Table 1.** Mechanistic roles of ACLY in kidney diseases.

Kidney diseases	Method of study	Model	Findings	References
Metabolic kidney disease	*In vivo*	ob/ob mice	↓ ACLY→ ↓ ACC, FAS, HMGCR → ↓ Ectopic lipid accumulation ↓Acetyl-CoA → ↓ Histone acetylation → Profibrotic transcription	[18]
	*In vitro*/*in vivo*	ob/ob BTBR mice; MCs	↑ TGF-β1 → ↑ RIPK3→ ↑AKT →↑ P-ACLY → Fibroblast Activation/Proliferation/ECM Production → Renal Fibrosis	[25]
	*In vitro*	MCs	↑ ACLY→ ↑ H3K9/14, H3K18 Ac → ↑ TGF-β1, TGF-β3, CTGF, FN and collagen type IV	[26]
	*In vitro*	Ob/+BTBR and ob/obBTBR mice; MCs	HG/P/TNF-α(NF-κB, PKA-CREB)→ ↑ ACLY→ ↑ ACC, FAS, ↑ HMGCR; H3K9/14 , H3K27 →↑ TGF-β1, FN	[27]
	*In vitro*/*in vivo*	HK-2 cells; VDR-KO and VDR-OE mice	↑ VDR→↓ ACLY →↓ROS, MDA → ↑ Nrf2/Keap1→ HO-1	[28]
Renal cell carcinoma	*In vitro*	ccRCC patient tumor tissue; 786-O and GRC-1 cells	↑ ACLY → ↑ ACC → ↑ Lipid Synthesis (*via* ACC1) , ↑ Histone Acetylation → Enhanced Cell Proliferation, Survival, Migration → Tumor Progression , Aggressiveness.	[29]
	*In vitro*/*in vivo* /clinical	Human RCC cell lines; BALB/c-Nude mouse; Tumor tissues and paired adjacent non-tumor tissues from ccRCC patients	HIF-2α →↑ LPCAT1→↓ NF-κB→↑ FBXW7→↓ ACLY → Reprogramming of Lipid Metabolism	[30]
	*In vitro*/*in vivo*	786-O and HepG2 cells; db/db mice	↓ pVHL→↑ PPARγ (stabilized) →↑ ACLY →↑ Lipogenesis →↑ Lipid Accumulation / ↑ Tumor Progression	[31]
	*In vitro*/ *in vivo*/clinical	ccRCC cell line; BALB/C nude mice; ccRCC tumor tissues	↓ GATA3 →↑ LINC00887 →↑ ACLY-BP →↑ ACLY Protein Level →↑ Acetyl-CoA Level →↑ Lipid Droplet Accumulation →↑ Tumor Growth	[32]
Hypocitraturia and renal stone diseases	*In vivo*	Male Sprague-Dawley rats	Chronic Acidosis / Hypokalemia → ↑ ACLY → Hypocitraturia	[33]
Polycystic kidney disease	*In vitro*	PT-derived and IMCD-derived Cells	Bempedoic Acid → ↑ FATP2 → ↓ BA-CoA→ ↓ ACLY , ↑ AMPK→ ↓ Acetyl-CoA, ↑ lipid synthesis, ↑ Fatty acid oxidation , ↓ mTOR/ERK signaling, ↑ Mitochondrial function→↑ renal function	[34]

ob/ob: Leptin Gene Knockout; ACC: acetyl-CoA carboxylase; FAS: fatty acid synthase; HMGCR: 3-hydroxy-3-methylglutaryl-CoA reductase; ob/+: wild-type littermate control; MC: mesangial cell; HG: high glucose; P: palmitate; TNF-α: tumor necrosis factor-alpha; NF-κB: nuclear factor kappa B; CREB: cAMP response element-binding protein; ACLY: ATP-citrate lyase; H3K9/14: histone H3 lysine 9 and 14; H3K27: histone H3 lysine 27; TGF-β1: transforming growth factor-beta 1; FN: fibronectin; H3K18Ac: histone H3 lysine 18 acetylation; CTGF: connective tissue growth factor; HK-2 cells: human kidney-2 cells; VDR: vitamin D receptor; ROS: reactive oxygen species; MDA: malondialdehyde; Nrf2: nuclear factor erythroid 2-related factor 2; Keap1: kelch-like ECH-associated protein 1; HO-1: heme oxygenase-1; RIPK3: receptor-interacting protein kinase 3; AKT: AKT serine/threonine kinase; ECM: extracellular matrix; 786-0 and GRC-1 cells: human renal carcinoma cell lines; ACC1: acetyl-CoA carboxylase 1; HIF-2α: hypoxia-Inducible Factor 2α; LPCAT1: lysophosphatidylcholine acyltransferase 1; FBXW7: F-Box and WD repeat domain containing 7; pVHL: protein Von Hippel–Lindau downregulated; PPARγ: peroxisome proliferator-activated receptor upregulated; GATA3: GATA binding protein 3; LINC00887: long intergenic non-protein coding RNA 887; PT-derived and IMCD-derived cells: proximal tubule-derived and inner medullary collecting duct-derived cells; FATP2: fatty acid transport protein 2 (also known as ACSVL1); mTOR/ERK: mechanistic target of rapamycin/extracellular signal-regulated kinase

## ACLY in kidney physiology

ACLY is a central metabolic enzyme that cleaves citrate to generate cytosolic acetyl-CoA, thereby bridging carbohydrate metabolism with *de novo* lipid synthesis and providing essential precursors for diverse cellular processes [22]. Beyond its well-established role in lipid metabolism and cellular signaling, emerging evidence implicates ACLY in renal development, primarily through its regulation of acetyl-CoA metabolism. Acetyl-CoA serves as a critical metabolic hub, influencing cell fate decisions, histone acetylation, and energy production [37]. During kidney development, the dynamics of nephron progenitor cells (NPCs) are crucial [38]. NPCs are a transient population that self-renews and differentiates to form nephrons, the kidney’s functional units responsible for filtration and homeostasis [39]. The final nephron number, determined largely by the size and behavior of the NPC pool, is a key determinant of long-term renal health, with low endowment being a risk factor for hypertension and kidney disease [40,41]. Research suggests ACLY influences NPC fate by modulating their metabolic state. For instance, younger, self-renewing NPCs exhibit a glycolytic bias, and ACLY-derived acetyl-CoA may support their proliferation and maintenance [42]. A pivotal study employing Six2-Cre-mediated knockout of ACLY in NPCs demonstrated severe developmental defects, including NPC depletion and reduced glomerular number, directly establishing ACLY’s essential role in NPC proliferation and differentiation [43,44]. Furthermore, ACLY activity is itself regulated by cellular nutrient status: it remains active during nutrient abundance to support growth and becomes inhibited during fasting, highlighting its role as a metabolic sensor in kidney developmental processes [43]. In summary, ACLY affects kidney development by modulating the acetyl-CoA pool, thereby influencing the metabolic programming, survival, and differentiation of NPCs.

## ACLY in DKD

DKD is a common complication in patients with both Types 1 and 2 diabetes mellitus. Its incidence peaks approximately 20 years after the onset of diabetes and ultimately affects 40–45% of diabetic patients [45]. In the United States and most developing countries, diabetic nephropathy is a significant factor contributing to end-stage kidney disease [46]. In the European Union, the number of people with stage 5 diabetic CKD is increasing by 3.2% annually [47]. DKD represents an irreversible nephropathy, with established pathological changes remaining unresponsive to current reversal strategies [48]. Currently, the primary therapeutic measures for DKD are reducing cardiovascular risk, controlling glycemia, regulating blood pressure, and inhibiting the renin–angiotensin system (RAS) [46]. Other emerging therapeutic approaches such as sodium-glucose cotransporter-2 inhibitor, Glucagon-like peptide-1, and dietary control have also received widespread attention [49]. Despite these advances, pharmacotherapies that directly target the dysregulated lipid metabolic pathways central to DKD pathogenesis remain notably absent from the current therapeutic arsenal.

ACLY is implicated in diabetic pathophysiology through its roles in both insulin secretion and hyperglycemia-induced renal injury. ACLY is highly expressed in human and rat pancreatic islets, and the enzyme activity is much higher than in the liver, suggesting its important role in insulin secretion [50]. A previous study has shown that inhibition of ACLY by either ACLY inhibitors or by shRNA induced β-cell apoptosis and ER stress [51]. In diabetes, persistent hyperglycemia serves as a key driver of complications such as DKD and directly modulates ACLY expression and localization, thereby linking metabolic disturbance to renal pathology [35]. In DKD, ACLY drives disease progression *via* multiple interconnected pathways. Previous studies have demonstrated that histone methylation promotes fibrosis and drives inflammation, thereby accelerating the progression of kidney disease. Furthermore, histone acetylation also plays a pivotal role in this process [52]. Under hyperglycemic conditions, ACLY not only upregulated but also translocated to the nucleus. There, it converts citrate to acetyl-CoA, providing substrate for histone acetylation and epigenetic upregulation of pro-fibrotic genes (e.g., TGFβ1, TGFβ3, and CTGF) [26]. This promotes glomerulosclerosis and enhances synthesis of extracellular matrix proteins such as fibronectin and collagen IV, accelerating renal fibrosis [53].

Concurrently, ACLY contributes to oxidative stress and lipid peroxidation in DKD. Studies demonstrate that under oxidative stress, dysregulated ACLY disrupts redox homeostasis [54,55]. Research indicates that kidney fibrosis is driven by multiple factors, with impaired fatty acid oxidation emerging as a significant contributor [56]. In vitamin D receptor (VDR)-deficient DKD models, ACLY expression increases, leading to accumulation of reactive oxygen species (ROS) and lipid peroxidation markers (MDA, 4-HNE) while depleting the Nrf2/Keap1 antioxidant system, thereby aggravating renal injury. Conversely, VDR activation reduces ACLY expression and mitigates cellular damage, indicating that ACLY functions as a key mediator within the VDR-regulated lipid peroxidation and cytoprotective pathway [28]. Collectively, these findings underscore a significant association between ACLY activity and both glucose homeostasis and DKD progression.

## ACLY in obesity related kidney injury

Obesity represents another major metabolic risk factor strongly associated with kidney disease, yet the precise mechanisms linking obesity to renal injury remain incompletely understood [57,58]. As a key driver of hypertension and diabetes, obesity significantly contributes to the progression of metabolic kidney diseases [59,60]. Ectopic lipid accumulation and chronic inflammation are widely recognized as central pathological processes in obesity-related renal damage [8,61]. Renal ectopic lipid accumulation occurs when excess free fatty acids released from adipose tissue are taken up by the kidney. These fatty acids are then esterified into triglycerides, leading to abnormal intracellular lipid deposition [62]. The enzyme ACLY, a critical node in *de novo* lipogenesis, emerges as a potential mechanistic link in this context. Notably, ACLY activity in the liver exhibits an inverse relationship with dietary fat intake in rats, with this response being more pronounced in younger animals. This observation has prompted investigation into its role in obesity-associated kidney injury [63]. In our recent study, ACLY was found to be highly expressed in the kidneys of overweight/obese patients with CKD and in ob/ob BTBR mice [27]. This elevated ACLY expression drives histone hyperacetylation (H3K9/14 and H3K27), leading to the transcriptional upregulation of key adipogenic enzymes such as acetyl-CoA carboxylase (ACC), fatty acid synthase, and HMG-CoA reductase [64]. Furthermore, ACLY-generated acetyl-CoA supplies the necessary substrate for these enzymes, thereby accelerating *de novo* lipogenesis and promoting ectopic lipid accumulation within renal tissues [27,35]. The resulting quantitative and qualitative lipid overload triggers lipotoxic injury through inflammation, oxidative stress, and mitochondrial dysfunction, ultimately manifesting as tubular damage, mesangial hyperplasia, and endothelial activation [65]. In glomerular mesangial cells, synergistic stimulation by hyperglycemia, palmitic acid, and TNF-α upregulates ACLY expression *via* the NF-κB and PKA pathways [26,27]. Importantly, pharmacological inhibition of ACLY under these conditions completely abrogates histone hyperacetylation and the induction of adipogenic and fibrogenic proteins [26].

## Role of ACLY in renal fibrosis

Renal fibrosis represents a key pathological feature of CKD and is closely associated with the progressive decline of renal function [66]. Evidence from both unilateral ureteral obstruction (UUO) and adenine diet‑induced renal fibrosis models supports that the AKT/ACLY signaling pathway plays an important role in renal fibrosis. The results showed that RIPK3 knockout mice exhibited attenuated renal fibrosis along with reduced phosphorylation levels of AKT and ACLY, suggesting that RIPK3 may promote fibrosis partly through activation of the AKT/ACLY pathway [25]. Furthermore, transforming growth factor‑β1 (TGF‑β1), a potent profibrotic mediator, drives extracellular matrix accumulation and myofibroblast differentiation; these effects are similarly suppressed upon inhibition of either AKT or ACLY [67]. Human genetic studies provide further evidence for the pathological relevance of ACLY. A Mendelian randomization study demonstrated a causal relationship between ACLY and CKD, revealing that a 34% reduction in genetically predicted ACLY expression was associated with a 9% lower risk of CKD [68]. Although no direct causal relationship was observed between ACLY and estimated glomerular filtration rate (eGFR) or urinary albumin-to-creatinine ratio, these collective findings underscore the potential of ACLY as a therapeutic target in kidney diseases [17,69]. In particular, ACLY inhibitors may hold promise for the prevention or treatment of the renal fibrosis in CKD, warranting further investigation in clinical trials.

## ACLY in renal cell carcinoma

ACLY has garnered significant attention in cancer research due to its central role in cellular metabolism [22]. It serves as a critical link between glycolysis and lipogenesis, catalyzing the conversion of citrate to acetyl-CoA, a key precursor for fatty acid and cholesterol synthesis. This metabolic reprogramming, characterized by abnormal glucose and lipid metabolism, is a hallmark of tumor cells [69]. In renal cell carcinoma (RCC), ACLY expression is significantly upregulated compared to adjacent normal renal tissues [70]. Functional studies demonstrate that knockdown of ACLY *via* small interfering RNA not only suppresses the proliferation and migration of RCC cells but also promotes their apoptosis [29], highlighting ACLY as a promising therapeutic target in oncology [71].

Among urological malignancies, RCC is one of the most common, with clear cell RCC (ccRCC) being the predominant histological subtype, accounting for approximately 75% of cases [72,73]. Pathologically, ccRCC is distinguished by cytoplasmic accumulation of lipids and glycogen, which confers the characteristic ‘clear’ appearance to the cells [74]. A seminal molecular feature of ccRCC is the frequent inactivation of the von Hippel–Lindau (VHL) tumor suppressor gene, located on chromosome 3p25 [75, 76]. The VHL protein functions as an E3 ubiquitin ligase that targets hypoxia-inducible factors (HIFs), notably HIF-1α and HIF-2α, for degradation under normoxic conditions [77,78]. Consequently, VHL loss leads to the constitutive stabilization of HIFs. While HIF-1α is often associated with tumor-suppressive functions, HIF-2α is a well-established driver of aggressive tumor growth in ccRCC [79]. The expression and activity of ACLY in ccRCC are regulated through multiple, interconnected mechanisms downstream of VHL inactivation. A key pathway involves the HIF-2α/LPCAT1/FBXW7 axis. HIF-2α directly binds to the promoter of LPCAT1, enhancing its transcription [30]. Knockdown of LPCAT1 inhibits ccRCC cell proliferation, migration, invasion, and lipid accumulation [80]. Mechanistically, LPCAT1 silencing activates the NF-κB pathway, which upregulates the E3 ubiquitin ligase FBXW7. FBXW7, in turn, interacts with and promotes the degradation of ACLY, thereby reducing fatty acid synthesis. Crucially, overexpression of ACLY can reverse the anti-tumor effects induced by LPCAT1 knockdown, both *in vitro* and *in vivo*, underscoring ACLY’s pivotal role in this regulatory network [30].

Beyond the HIF-2α axis, VHL deficiency also promotes ACLY expression *via* HIF-independent pathways. For instance, VHL loss enhances the stability of peroxisome proliferator-activated receptor gamma (PPARγ), a nuclear receptor that transcriptionally activates the ACLY gene, thereby fueling tumor cell proliferation [81,82]. PPARγ is a master regulator of adipogenesis and lipid metabolism [31]. Additionally, post-translational stabilization of ACLY contributes to its oncogenic function. The micropeptide ACLY-BP, encoded by the LINC00887 gene (which is suppressed by GATA3), stabilizes ACLY by maintaining its acetylation and inhibiting its ubiquitin-mediated degradation. This stabilization leads to enhanced lipid deposition and stimulates ccRCC cell proliferation [32]. Collectively, these findings illustrate that ACLY expression in ccRCC is orchestrated by a multifaceted regulatory network involving transcriptional activation, protein stabilization, and altered degradation.

The expression level of ACLY is closely associated with tumor prognosis. Research indicates that ACLY may modulate tumor cell activity through pathways such as the mammalian target of rapamycin complex 1 (mTORC1) and appears to correlate with immune infiltration patterns, suggesting its potential value as a prognostic biomarker in RCC [70]. However, the precise mechanisms by which ACLY influences tumor progression and patient outcomes require further elucidation. More extensive research and clinical validation are necessary to determine whether ACLY can reliably serve as a diagnostic or prognostic marker in clinical practice. In summary, ACLY occupies a central node in the metabolic and signaling landscape of ccRCC, making it a compelling target for therapeutic intervention.

## ACLY in hypocitraturia and renal stone diseases

Renal stone disease, also known as nephrolithiasis, is a common disease affecting approximately one in every eleven people in the United States (around 9%). By the age of seventy, the lifetime prevalance approaches 20% among men [83]. The causes of renal stone disease are numerous, such as dietary factors, climate factors, and genetic factors. Renal stone disease, as a common disease of the urinary system, brings a heavy burden to individuals and society. Hypocitraturia is an important factor in renal stone formation. This metabolic disorder has become an effective clinical strategy for the prevention and treatment of renal stone disease. At the same time, a strong relationship exists between citrate uptake and utilization and ACLY activity in the kidney.

Hypocitraturia occurs in about 50% of patients with renal stone disease [84]. Citrate can bind calcium ions in the gastrointestinal tract, which in turn reduces intestinal absorption of calcium, lowers the concentration of calcium in the urine. This reduction in urinary calcium reduces the deposition of calcium salts in the kidneys, and plays an important role in the prevention of calcium oxalate and other calcium-based stone formation [85]. Under appropriate pH, the solubility of certain stone components will increase, reducing the possibility of crystal precipitation, citrate can alkalize the urine, thus reducing the risk of stone formation [86]. Factors contributing to hypocitraturia may be related to age, metabolic acidosis, and hypokalemia.

ACLY plays a key role in the pathogenesis of hypocitraturia. Under physiological conditions, renal proximal tubules (PTs) are responsible for the reabsorption and metabolism of citrate, and ACLY is a key enzyme involved in the regulation of this process. It has been shown that chronic metabolic acidosis leads to significant physiological changes in rats, which in turn lead to the development of hypocitraturia, with a dramatic increase in the activity and protein amount of ACLY in the renal cortex after 7 days, but not in the amount of mRNA [33]. Potassium deficiency also contributes to hypocitraturia and altered ACLY activity. Potassium deficiency in the body causes intracellular acidosis, which disrupts citrate metabolism and promotes an increase in ACLY activity in the cytoplasm. This results in a decrease in the citrate content of renal tubule cells, and in response to this change, tubule cells increase reabsorption of filtered citrate, resulting in lower urinary citrate content [87]. The activity of ACLY was higher in young mice than in adult mice, and further studies are needed to determine whether this is related to the development of kidney stones [88].

However, the existing experimental evidence predominantly stems from mouse models. Further data are necessary to elucidate the mechanisms of ACLY in humans and to determine whether it can be regarded as a novel therapeutic approach for kidney stones. Overall, ACLY is intimately connected with low citrate urine levels. Variations in its activity and protein abundance significantly influence urinary citrate excretion. A thorough investigation into this relationship is crucial for advancing our understanding and treatment of related disorders, including renal stone disease.

## ACLY in polycystic kidney disease

The most common type of polycystic kidney disease is autosomal dominant polycystic kidney disease (ADPKD). ADPKD is an autosomal dominant type of polycystic kidney, one of the most common genetic disorders leading to end-stage kidney disease and kidney failure [89]. The feature of ADPKD is the progressive, bilateral development and enlargement of focal cysts [90]. There are few treatments for ADPKD, and the main drug is tolvaptan, but it has significant side effects [91]. It has been suggested that ADPKD is a metabolic disease associated with mitochondrial dysfunction [92].

Most ADPKD patients have deletions or mutations in the PKD1 and PKD2 genes [93]. Patients with pkd1 deletion mutations tend to have more severe disease, and multiple cysts often form in the kidneys of patients, eventually leading to ESRD [90]. In the kidneys of pkd1 knockout mice, a study found that ACLY activity was upregulated. Transcriptional profiling, metabolomics, and lipidomics analyses revealed dysregulation of metabolic pathways in the kidneys of mutant mice, with significant changes in lipid metabolism pathways, suggesting that ADPKD may be an altered cellular metabolic disease [94]. In PT and medullary collecting duct (IMCD) derived Pkd1 knockout cells, ACLY activity was increased compared to control parental cell lines [34]. This suggests that elevated ACLY activity may be associated with the cyst-forming ability of ADPKD epithelial cells and that its higher activity may promote cystic growth and disease progression in polycystic kidneys. Besides, in this research, it was found that the ACLY inhibitors bempedoic acid and SB-204990 significantly inhibited the cystic growth of PT and IMCD-derived Pkd1 knockout renal cell lines under 3D conditions, and the therapeutic effect was similar to that of tolvaptan, suggesting that the inhibition of ACLY may be helpful for alleviating the symptoms of ADPKD, and the combination of these two inhibitors also suggests a new idea for the subsequent treatment of ADPKD [34]. A clinical trial of an ACLY inhibitor for kidney disease is currently underway. This is a phase II, randomized, double-blind, placebo-controlled, multicenter trial (BEAT-PKD; NCT07282821) for ADPKD. The study aims to enroll 120 patients with ADPKD at risk of rapid disease progression. It seeks to evaluate the safety and tolerability of bempedoic acid or placebo treatment, with the potential to reverse metabolic dysfunction in ADPKD patients and thereby delay disease progression.

At present, research on ACLY in the field of polycystic kidney disease remains at a preliminary stage. Given the key role of ACLY in lipid metabolism and the close association between the pathogenesis of polycystic kidney disease and metabolic dysregulation, thus, investigating the role of lipid metabolism may provide a strategic entry point to elucidate the link between ACLY and ADPKD pathogenesis. Further exploration in this direction could provide valuable insights into the underlying mechanisms and potential therapeutic strategies.

## ACLY as a target in kidney diseases

**Figure 2.**
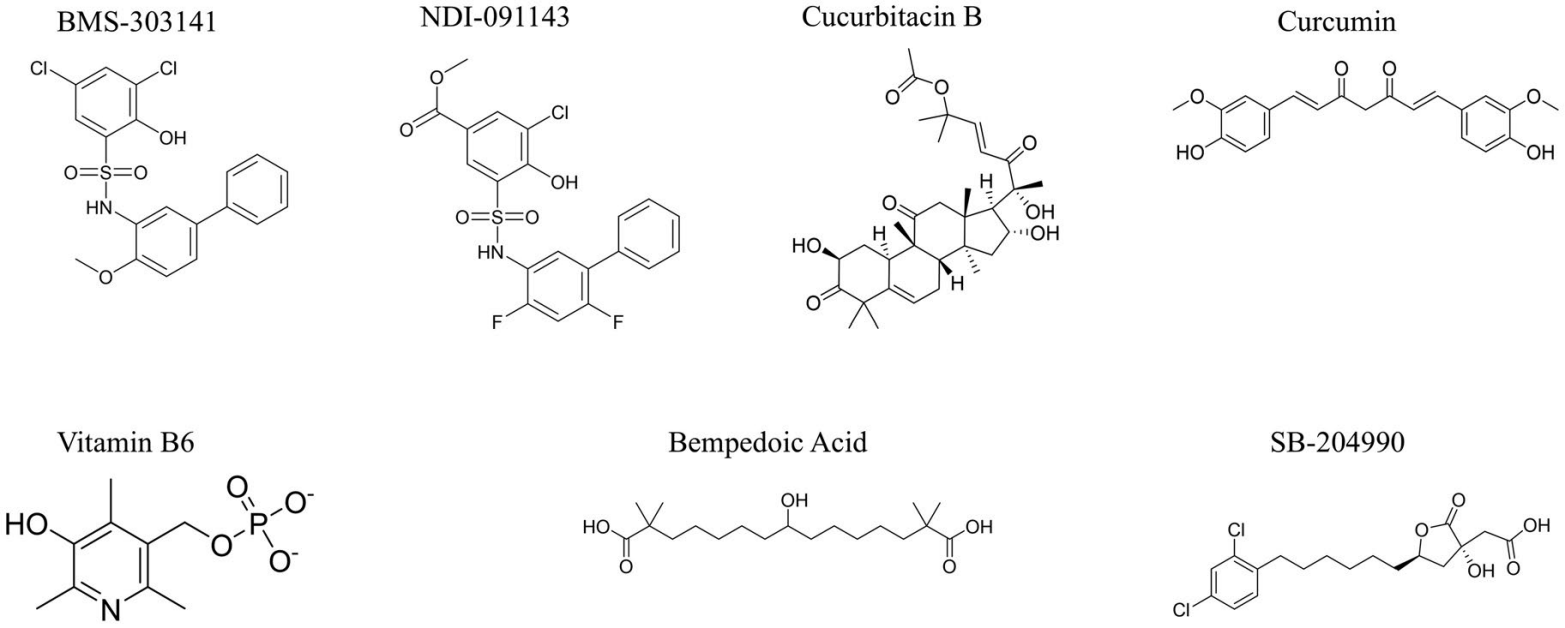
Chemical structures of four ACLY inhibitors (Bempedoic acid, SB-204990, BMS-303141 and NDI-091143). The chemical structure information was obtained from PubChem (https://pubchem.ncbi.nlm.nih.gov).

So far, few therapeutic studies have been conducted on ACLY in kidney diseases. However, several ACLY inhibitors have been found to play important roles in reducing lipid metabolism and inhibiting tumor cell growth. In the following sections, we will provide a detailed introduction to several small molecule inhibitors of ACLY as well as natural product inhibitors (Figure 2 and Table 2). This aims to offer a valuable reference for advancing research on ACLY in the treatment of kidney diseases.

**Table 2.** Clinical and basic research of ACLY inhibitors.

Inhibitors	Stage of development	Model	Effects	References
Bempedoic -acid	Clinical	Patients with high cardiovascular risk and statin intolerance	Reduce the cardiovascular risk of patient intolerant to statins	[95]
	*In vitro*/*in vivo*	Primary rat hepatocytes; HepG2 human hepatoma cell line; Golden Syrian hamsters; C57BL/6N mice	Direct inhibition of ACLY thereby suppresses fatty acid and cholesterol synthesis	[96]
	Clinical	Subjects with renal impairment stratified as mild, moderate, or severe	No dose adjustment is required for patients with mild to moderate renal impairment	[97]
	*In vivo*	12-week-old db/db mice	By inhibiting the expression of renal lipogenic enzymes, reducing ectopic lipid accumulation, and alleviating inflammatory responses and fibrotic processes	[18]
SB-204990	*In vitro*/*in vivo*	Hep G2 cells; Sprague-Dawley rats; Beagle dogs	Inhibiting hepatic VLDL synthesis and potentially upregulating LDL receptor activity, thereby lowering plasma cholesterol and triglyceride levels.	[98]
	*In vivo*	Wild-type male C57BL/6 mice; Primary Hepatocytes; AML12 Hepatocyte Cell Line; Primary Pancreatic Islets	Mediates cholesterol reduction and decreased hepatic lipid *via* AMPK interaction	[99]
NDI-091143	*In vitro*/*in vivo*	CAL27 cells; patient-derived cancer-associated fibroblasts (CAFs); Subcutaneous CAFs-enriched CAL27 tumor-bearing mice; CAFs-enriched OSCC tumor-bearing mice	Remodeling the tumor stroma to potentiate chemotherapy	[100]
BMS-303141	*In vivo*	BALB/c nude mice injected with HepG2 cells	Induces apoptosis in HCC cells *via* triggering ER Stress and activating the p-eIF2α/ATF4/CHOP axis	[101]
Vitamin B6	*In vivo*	Charles River CD rats	Vitamin B_6_ deficiency Significantly increased urinary oxalate excretion and reduced ACLY activity	[102]
Cucurbitacin B	*In vivo*/*in vitro*	PC-3 and LNCaP cells; Male athymic mice subcutaneously implanted with PC-3 cells	ACLY knockdown enhances, and its overexpression attenuates, CuB-induced apoptosis	[103]
Curcumin	*In vivo*	Male Wistar rats	Inhibits the expression of ACLY, thereby suppressing lipogenesis.	[104]

## Bempedoic acid

Elevated low-density lipoprotein cholesterol (LDL-C) is a well-established risk factor for atherosclerotic cardiovascular disease [105]. Statins remain the cornerstone of pharmacologic LDL-C lowering; however, their utility is limited by statin intolerance in a significant subset of patients and by persistent residual cardiovascular risk in others. These limitations underscore the need for effective and well-tolerated alternative therapies [106]. Notably, Mendelian randomization studies suggest that inhibitors of ACLY, a key enzyme in cholesterol biosynthesis, share a genetic mechanism of action with statins in reducing serum LDL-C levels [107]. Bempedoic acid (also known as ETC-1002) is a first-in-class, oral, non-statin LDL-C-lowering agent that functions as a selective ACLY inhibitor [108]. Approved by the U.S. FDA in 2020, it represents a significant advancement in the clinical management of hypercholesterolemia, particularly for statin-intolerant patients [109]. Its efficacy and safety profile are supported by robust clinical evidence. A large-scale, randomized trial involving 13,970 statin-intolerant patients demonstrated that bempedoic acid significantly reduced the risk of major adverse cardiovascular events [95]. Furthermore, a study of 2230 patients with atherosclerotic cardiovascular disease and hypercholesterolemia confirmed that adding bempedoic acid to maximally tolerated statin therapy significantly lowered LDL-C levels without increasing the overall rate of adverse events compared to placebo [110].

Bempedoic acid is a prodrug designed for targeted hepatic activation [111]. Following intestinal absorption, it is selectively converted to its active CoA form (bempedoic acid CoA) in the liver by the enzyme very-long-chain acyl-CoA synthetase 1 (ACSVL1). This activation is spatially restricted, as ACSVL1 is predominantly expressed in hepatocytes and absent in skeletal muscle and intestinal tissue, a property that may help avoid myopathic side effects associated with statins [112]. The active metabolite acts as a potent, high-affinity, and concentration-dependent inhibitor of ACLY, with a reported half-maximal inhibitory concentration (IC_50_) of 17.8 μM [96,113]. Pharmacokinetic studies indicate that bempedoic acid is well-tolerated across varying degrees of renal impairment, and no dose adjustment is required for patients with mild to moderate renal dysfunction [97]. Beyond its primary lipid-lowering role, emerging preclinical data suggest potential renoprotective effects. In a db/db mouse model of metabolic dysfunction, bempedoic acid demonstrated superior efficacy among ACLY inhibitors in ameliorating renal injury, inflammation, and fibrosis [18]. Its mechanism may extend beyond ACLY inhibition, as evidence indicates it can also regulate AMP-activated protein kinase (AMPK) activity, suggesting a dual-pathway modulation of cellular metabolism that may enhance its therapeutic effects [96].

Given the established link between aberrant renal lipid metabolism (ectopic lipid accumulation) and the pathogenesis of diabetic nephropathy and obesity-related kidney injury, the potent lipid-modifying action of bempedoic acid presents a compelling therapeutic hypothesis for these conditions. While its direct role in kidney diseases remains largely unexplored in clinical settings, its mechanistic profile and preclinical findings warrant further investigation. Substantial preclinical and clinical research is necessary to validate its potential efficacy and safety in treating renal pathologies associated with metabolic dysregulation (Table 3).

**Table 3.** Summary of the clinical trials of bempedoic-acid (ETC1002).

Inhibitor	NCT identifier	Clinical trial phase	Clinical indication	Status
Bempedoic -acid(ETC1002)	NCT07282821	Phase 2	Polycystic kidney disease	Not yet recruiting
	NCT03067441	Phase 3	Hypercholesterolemia	Completed
	NCT05694260	Phase 2	Hypercholesterolemia	Completed
	NCT06035874	Not applicable	Type 2 diabetes	Recruiting
	NCT02991118	Phase 3	Hypercholesterolemia	Completed
	NCT06780410	Phase 3	Hypercholesterolemia	Recruiting
	NCT06021951	Phase 4	Healthy lactating women	Completed
	NCT06686615	Observational	Mixed dyslipidemia	Recruiting
	NCT05103254	Observational	Pregnancy; hyperlipidemias	Recruiting
	NCT02988115	Phase 3	Hypercholesterolemia; Atherosclerotic Cardiovascular Disease	Completed
	NCT05488431	Phase 2	Dyslipidemias; Cardiovascular Diseases	Recruiting
	NCT03337308	Phase 3	Hyperlipidemias	Completed
	NCT03001076	Phase 3	Hypercholesterolemia; Atherosclerosis	Completed
	NCT06923956	Phase 1	Hypercholesterolemia; Mixed dyslipidemia	Completed
	NCT06925100	Phase 4	Primary Hypercholesterolemia or Mixed Hyperlipidemia	Completed
	NCT03193047	Phase 2	Hypercholesterolemia	Completed
	NCT04784442	Phase 2	Hypercholesterolemia	Completed
	NCT06686615	Observational	Primary hypercholesterolemiia	Recruiting
	NCT02666664	Phase 3	Hypercholesterolemia; atherosclerotic cardiovascular diseases	Completed
	NCT02659397	Phase 2	Hyperlipidemia	Completed
	NCT05263778	Phase 4	Cardiovascular diseases; NSTEMI	Unknown
	NCT06742853	Phase 1	Dyslipidemia	Completed
	NCT06450366	Phase 3	Hypercholesterolemia	Completed
	NCT05687071	Phase 3	Hyper-low-density Lipoprotein (LDL) cholesterolemia	Completed
	NCT05683340	Phase 3	Hyper-low-density Lipoprotein (LDL) cholesterolemia	Completed
	NCT02993406	Phase 3	Cardiovascular diseases; statin adverse reaction	Completed
	NCT05798390	Observational	Primary hypercholesterolemia; mixed dyslipidemia	Withdrawn
	NCT03531905	Phase 3	Diabetes mellitus, Type 2, cholesterolemia	Completed
	NCT02993406	Phase 3	Cardiovascular disease	Completed

## SB-204990

Beyond its established role in lipid metabolism, ACLY has garnered significant interest in oncology due to its crucial function in cancer cell metabolism and its potential as a therapeutic target. ACLY is pivotal for fueling tumor growth, influencing cancer cell proliferation, apoptosis, and stemness, with its expression levels often correlating with patient prognosis [114]. Consequently, the development of ACLY inhibitors represents a promising strategy for novel anticancer therapies. Among these investigational agents, SB-204990 is a potent, cell-permeable prodrug inhibitor of ACLY [98]. Its anticancer activity stems from a dual mechanism: the suppression of *de novo* lipid synthesis and the direct inhibition of cancer cell growth. *In vivo* studies demonstrate its capacity to induce tumor differentiation, while *in vitro* evidence confirms its role in impairing aerobic glycolysis (the Warburg effect) in cancer cells [99,115]. Further multi-omics analyses have elucidated that SB-204990 modulates a network of molecular pathways associated with aging and tumorigenesis. These include central regulators of cellular metabolism and growth such as energy metabolism, mitochondrial function, the mTOR signaling pathway, and the folate cycle, all of which are critically involved in sustaining tumor progression [99].

## NDI-091143

NDI-091143 also is a small molecule inhibition of ACLY. The structure of NDI-091143 is observed by an electron microscope. NDI-091143 is a 2-hydroxy-N-arylbenzenesulfonamide analog that has a benzenesulfonamide core structure and contains a polar portion (phenol ring) and a major hydrophobic portion (2,4-difluorobiphenyl group) [116]. NDI-091143 binds in a metastable, predominantly hydrophobic cavity near the citrate-binding site of ACLY, and its binding leads to extensive conformational changes in the citrate domain of ACLY, changes that indirectly block citrate binding and recognition [117]. Based on NDI-091143, a study has identified a novel macrocyclic ACLY inhibitor 2 with increased stability, a new drug that deserves further investigation. Just like SB-204990, NDI-091143 also plays roles in cancer cells. Combined with chemotherapeutic agents (e.g., adriamycin DOX or paclitaxel PTX), it can act on both tumor-associated fibroblasts and tumor cells by self-assembling to form a carrier-free nano-agent (CFNA), which enhances the chemotherapeutic effect [100].

## BMS-303141

As an investigational ACLY inhibitor, BMS303141 has demonstrated significant biological activities across different pathological contexts, highlighting its multifunctional therapeutic potential. In oncology, BMS303141 exhibits promising antitumor effects. A study in hepatocellular carcinoma (HCC) revealed that BMS303141 induces apoptosis in HCC cells by triggering endoplasmic reticulum stress and activating the p-eIF2α/ATF4/CHOP signaling axis [101]. Notably, it showed a synergistic effect, significantly enhancing the therapeutic efficacy of the standard-of-care agent sorafenib. Beyond cancer, BMS303141 has shown efficacy in metabolic bone disease. Research on osteoporosis demonstrated that this inhibitor prevented ovariectomy (OVX)-induced bone loss *in vivo*. The mechanism involves the suppression of osteoclast differentiation and function, mediated in part through the regulation of histone acetylation [118]. This study further identified Rac1 as a critical downstream regulator of ACLY in the osteoclast differentiation pathway. Collectively, these findings position BMS303141 not only as a potential adjunct in cancer therapy but also as a promising candidate for the treatment of conditions like osteoporosis, underscoring the broad therapeutic relevance of targeting ACLY.

## ACLY inhibitors derived from natural sources

In addition to synthetic compounds, numerous naturally occurring molecules have been identified as modulators of ACLY activity, offering a rich resource for drug discovery and mechanistic studies across various diseases.

Vitamin B6, an essential coenzyme involved in numerous metabolic and signaling pathways, influences ACLY activity. Experimental studies in rats have shown that vitamin B6 deficiency leads to decreased hepatic ACLY activity, potentially linked to reduced insulin levels [102,119]. Its recognized role in managing urinary stones may also be connected to this modulation of ACLY function [120].

Cucurbitacin B (CuB), a triterpenoid found in plants of the *Cucurbitaceae* family such as *Cucurbita pepo*, possesses notable anticancer properties [121]. In a prostate cancer mouse model, CuB treatment reduced tumor size by nearly 80%. This pro-apoptotic effect was abrogated by ACLY overexpression, confirming that inhibition of the ACLY signaling pathway constitutes a key anti-tumor mechanism of CuB [103].

Curcumin, the principal bioactive polyphenol in turmeric (*Curcuma longa*), is renowned for its anti-inflammatory and anticancer effects [122]. In an animal study, curcumin was found to suppress ACLY expression in rats fed a high-fructose diet, thereby helping prevent fructose-induced hyperlipidemia [104]. This suggests its potential utility in mitigating metabolic complications, including those associated with diabetic nephropathy, beyond glycemic control.

Hydroxycitric acid (HCA), derived from *Garcinia cambogia*, acts as a competitive ACLY inhibitor [123]. By depleting acetyl-CoA, it inhibits protein acetylation and promotes autophagy [124]. In breast cancer, HCA has been shown to re-sensitize tamoxifen-resistant cells to treatment [125]. Furthermore, it inhibits lipogenesis by downregulating ACLY, SREBP-1c, and FAS expression, while simultaneously promoting lipolysis and reducing lipid deposition *via* increased hepatic lipase activity and PPARα expression [126].

Radicicol, a natural macrocyclic antibiotic, functions as a noncompetitive ACLY inhibitor and impedes cell migration under glycolytic conditions [127]. It also attenuates glucose- and GLP-1-stimulated insulin secretion in pancreatic models, indicating that ACLY inhibition can disrupt normal insulin release regulation [128]. However, further validation in human pancreatic islets is required.

In summary, ACLY holds great promise as a novel target across numerous medical fields. Various ACLY inhibitors have emerged and have been proved to be not only highly effective but also relatively safe. However, research of these inhibitions on ACLY treatment on kidney diseases remains limited at present. We eagerly anticipate the emergence of more relevant studies in this area.

## Conclusions and perspectives

**Figure 3.**
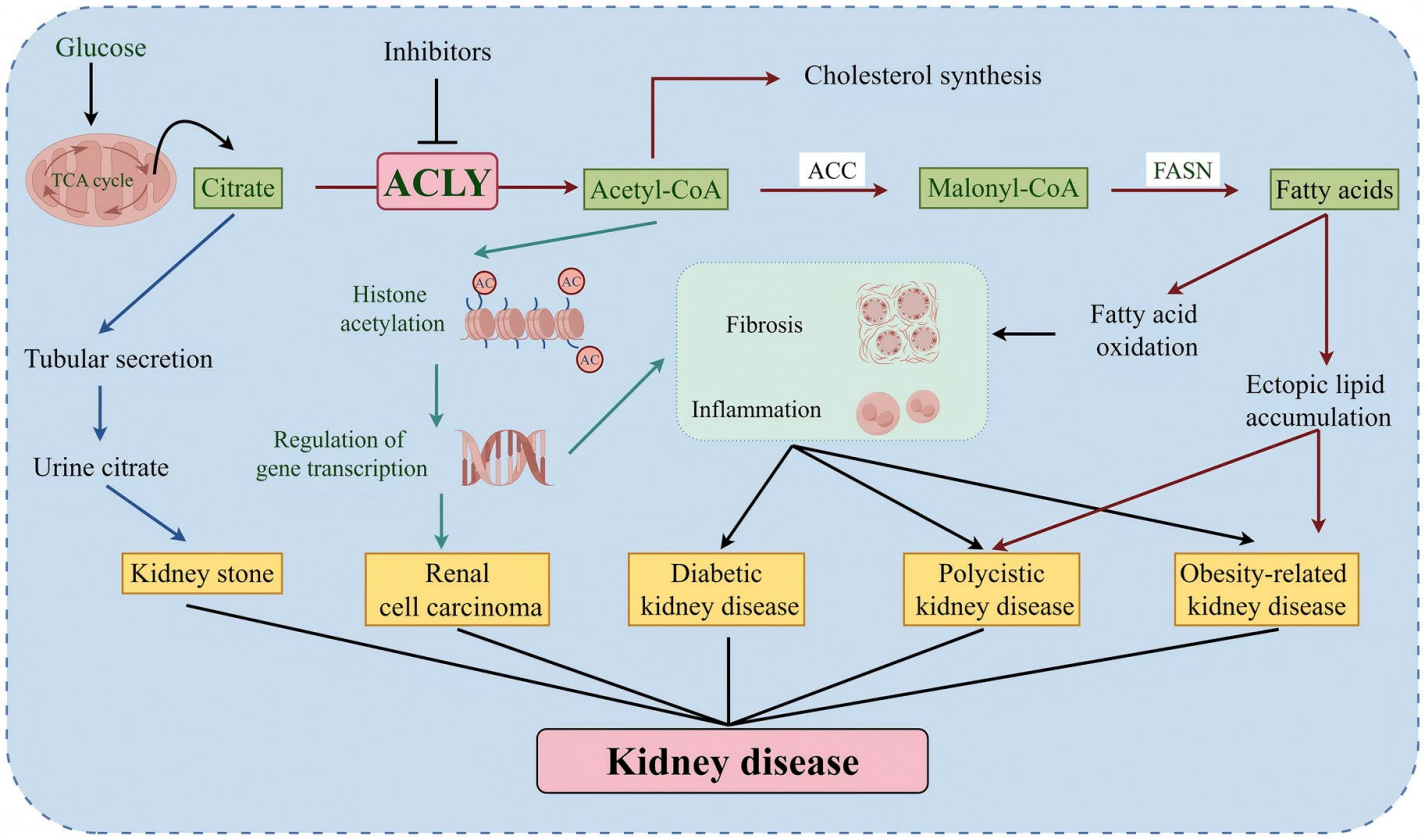
Regulatory mechanisms of ACLY in the physiology and pathophysiology of the kidney. Glucose undergoes the tricarboxylic acid cycle to produce citrate, which is subsequently transported from mitochondria to the cytoplasm and catalyzed by ACLY to form acetyl-CoA. As a pivotal metabolic intermediate, acetyl-CoA exerts central functions through two pathways. On the one hand, it forms acetyl-CoA carboxylase-generated malonyl-CoA, which is catalyzed by fatty acid synthase to synthesize fatty acids, leading to intracellular ectopic lipid deposition. On the other hand, it participates as an acetyl donor in histone acetylation modifications, regulating gene transcription and thereby influencing cellular function and phenotype. Furthermore, reduced urinary citrate excretion is closely associated with the development of renal calculi. These metabolic-epigenetic pathways play significant roles in multiple renal disorders, including RCC, diabetic nephropathy, polycystic kidney disease, and obesity-related nephropathy. This suggests that targeted inhibition of these pathways may offer novel therapeutic targets and intervention strategies for renal diseases. This figure was generated by Figdraw (www.figdraw.com).

In summary, ACLY emerges as a central metabolic and epigenetic integrator that critically underpins the pathophysiology of a remarkably broad spectrum of kidney diseases. Beyond its canonical role in *de novo* lipogenesis, ACLY bridges nutrient sensing, cellular signaling, and gene regulation by governing the availability of acetyl-CoA – a key substrate for both lipid synthesis and histone acetylation. This review consolidates compelling evidence that dysregulation of ACLY is not a mere bystander but a pathogenic driver in DKD, obesity-related renal injury, renal fibrosis, ccRCC, hypocitraturia-related nephrolithiasis, and polycystic kidney disease (Figure 3). Mechanistically, ACLY exerts its detrimental effects through diverse yet interconnected pathways, including the AKT/ACLY axis in fibrosis, the HIF-2α/LPCAT1/FBXW7 and VHL/PPARγ networks in ccRCC, and the promotion of histone hyperacetylation and lipotoxicity in metabolic kidney diseases. The convergent role of ACLY across these disparate conditions positions it as a singular, promising therapeutic target. Encouragingly, several pharmacological inhibitors – including the FDA-approved bempedoic acid, as well as SB-204990, NDI-091143, and various natural compounds – have demonstrated efficacy in preclinical models by attenuating lipid accumulation, fibrosis, inflammation, and tumor growth. Mendelian randomization studies further lend genetic support for its causal involvement in CKD risk. However, despite these promising advances, the translation of ACLY-targeted therapies into renal clinical practice remains nascent.

Looking forward, several key perspectives and challenges warrant emphasis. First, future research must move beyond association to dissect the precise, cell-type-specific mechanisms by which ACLY contributes to disease progression. The differential roles of cytosolic *versus* nuclear ACLY pools, and their interaction with cell-specific signaling networks (e.g., in podocytes, tubular cells, or fibroblasts), require deeper exploration. Second, while inhibitors like bempedoic acid show promise for cardiovascular lipid lowering, their direct renal protective effects, optimal dosing, and long-term safety profiles in patients with kidney disease demand rigorous evaluation in dedicated preclinical and clinical trials. Third, the potential of ACLY as a diagnostic or prognostic biomarker across various renal pathologies should be systematically validated in large, diverse patient cohorts. Fourth, combination therapies represent a strategic avenue to enhance efficacy and overcome potential resistance. These approaches integrate ACLY inhibition with existing standard-of-care agents, including SGLT2 inhibitors, RAS blockers, or chemotherapeutics. Finally, emerging technologies such as single-cell multi-omics, spatial metabolomics, and advanced molecular imaging will be instrumental in mapping the dynamic activity and regulation of ACLY within the complex architecture of the diseased kidney.

In conclusion, ACLY stands at a critical nexus linking metabolism, epigenetics, and renal pathology. Targeting this enzyme offers a novel and unifying therapeutic strategy with the potential to address multiple unmet needs in nephrology. A concerted effort to bridge mechanistic understanding with clinical translation will be essential to realize the full promise of ACLY-directed therapeutics in improving outcomes for patients with kidney disease. Therefore, given the availability of existing drugs such as Bempedoic acid, it is undoubtedly the logical next step to conduct prospective clinical trials specifically evaluating the efficacy of ACLY inhibitors in the kidney disease.

## CRediT authorship contribution statement

Mengjiao Wei: Writing – review & editing, Writing – original draft, Visualization, Investigation, Conceptualization. Mina Tao: Writing – review & editing, Investigation. Huifang Tan: Writing – review & editing. Zhiyuan Jin: Writing – review & editing. Yiya Yang: Investigation, Funding acquisition. Zheng Xiao: Investigation, Funding acquisition. Guoli Li: Writing – review & editing, Supervision, Funding acquisition. Yinyin Chen: Writing – review & editing, Supervision, Funding acquisition.

## Data Availability

Data sharing is not applicable to this Review as no new data were created or analyzed in this article.
